# Hygienic Practices of Vendors and Their Contribution to Coliform, *Salmonella*, and *Shigella* Bacteria of Raw Milk at Asella Town, Oromia, Ethiopia

**DOI:** 10.1155/2024/8869022

**Published:** 2024-02-27

**Authors:** Hirpo Tusa, Tsegaye Alemayehu, Bereket Wake Subussa, Henok Ayalew, Musa Mohammed Ali

**Affiliations:** ^1^Arsi University College of Health Sciences, Asella, Oromia Regional State, Ethiopia; ^2^Hawassa University, College of Medicine and Health Sciences, School of Medical Laboratory Science, Hawassa, Sidama Regional State, Ethiopia; ^3^St. Paul's Hospital Millennium Medical College, Addis Ababa, Ethiopia

## Abstract

**Background:**

Coliform, *Salmonella*, and *Shigella* are among the most encountered bacteria in raw milk. This study is aimed at determining the extent of coliform, *Salmonella*, and *Shigella bacteria* in raw milk and vendor hygiene practices at Asella town, Oromia Regional State, Ethiopia, from March 1 to 30, 2022.

**Methods:**

In this study, 210 milk vendors were included; each vendor provided a 50 ml sample of raw milk. Bacteria were isolated and identified using standard bacteriological techniques. Data were entered and analyzed using EPI info version 7 and SPSS version 22, respectively. A binary logistic regression model was applied to determine the factors associated with bacterial contamination of raw milk.

**Results:**

The total contamination percentage of raw milk was 50 (23.8%) (95% CI: 18.1-29.5%). The predominant bacteria identified were coliform 43 (20.5%) followed by *Salmonella* species 7 (3.3%). Among coliforms, the predominant bacteria were *Citrobacter* species 15 (34.9%) followed by *Enterobacter* species 11 (25.6%), *Escherichia coli* and *Serratia* species each 6 (14%), and *Klebsiella* species 5 (11.6%). However, no *Shigella* was isolated in this study. Not having the habit of washing cow teats (*p* < 0.0001), the habit of washing teats with tap water (*p* < 0.0001), not having separate cloth during milking (*p* < 0.0001), not having a practice of testing milk for bacterial contamination (*p* = 0.027), and not having separate vending environment (*p* = 0.039) were significantly associated with bacterial contamination of raw milk.

**Conclusions:**

The percentage of bacterial contamination of milk was found to be high. Participants without a habit of washing cow teats, a habit of washing milk utensils with only tap water, and not having separate vending environments were associated factors for bacterial contamination of raw milk. Milk vendors are advised to develop the habit of washing teats before milking, avoid washing teat/milk utensils only with tap water, and have a separate vending environment.

## 1. Introduction

Milk is a unique food that has long provided people with nourishments [1] that contains more readily digested nutrients than any other single food, including proteins, lipids, carbs, vitamins, and minerals, and it also provides immunogenic protection [1, 2]. Due to its significance, milk is known as “white gold” [3]. As things stand, the establishments in these economies that sell milk and milk products are not sufficiently regulated or overseen by the appropriate regulatory bodies, and they maintain unsanitary conditions [4].

Several factors, such as the animal's health, farm management techniques, environmental hygiene, and inadequate temperature control, influence the microbiological status of raw milk [5]. It is difficult to maintain premium, high-quality milk from a nearby farm for an open market because of adulteration, humidity, dangerous food chains, and unsanitary farmer milking practices. Unsanitary methods and subpar animal care on farms put farmers, customers, and the public at risk of bacterial resistance and other illnesses linked to milk [6].

Due to its high water content, nearly neutral pH, and variety of available essential nutrients, milk is an excellent growth medium for a wide range of microorganisms [7]. Fresh milk typically has a low microbial count (less than 1000 CFU/ml^−1^ of milk); it is aseptically extracted from clean, healthy cows. From the moment it leaves the cow's teat until it is consumed, it absorbs many microbes [8]. *Yersinia enterocolitica*, *Shigella*, *Listeria monocytogenes*, coliforms, *Salmonella Typhimurium*, *Campylobacter jejuni*, *Bacillus cereus*, *Escherichia coli* 0157:H7, *Mycobacterium tuberculosis*, *Mycobacterium bovis*, and *Staphylococcus aureus* have all been linked to foodborne outbreaks linked to the consumption of raw milk, according to various studies [7, 9–11].

Even though raw milk can harbor multiple pathogens, this study focuses on *Shigella*, *Salmonella*, and coliform. Coliforms are rods that are non-spore-forming, Gram-negative, aerobic, or facultative anaerobic, and that can ferment lactose at a temperature of 32 to 35°C by producing gas and acid. It contains the following genera: *Escherichia*, *Enterobacter*, *Citrobacter*, and *Klebsiella* [12, 13]. Many different pathogens can cause foodborne illnesses; among them, *Salmonella* is thought to be the most common globally and has long been known to be a significant zoonotic bacterium that affects both humans and animals and is particularly important in developing nations like Ethiopia [14]. However, in high-risk groups like children, the elderly, toddlers in daycare centers, and patients in custodial institutions, *Shigella* continues to be the cause of mortality and/or morbidity [15].

In Ethiopia, it is customary to consume raw milk and its byproducts, which is unsafe for consumers' health because it can spread several diseases. Fresh milk is sold to the public by cooperatives of dairy farmers, informal markets, or small producers directly, which has presented a significant challenge to milk quality control on all fronts [16]. Furthermore, hygiene inspections of milk and milk products are not typically carried out regularly in Ethiopia. Aside from this, door-to-door delivery of raw milk in urban and periurban areas is frequently done with almost no quality control at any stage [17]. Data regarding the microbial profile of raw milk is, nevertheless, scarce in the majority of Asella town's milk vendors. Finding out the amount of *Shigella*, *Salmonella*, and coliform in raw milk as well as the vendors' hygienic practices in Asella town, Oromia, Ethiopia, was the goal of this study.

## 2. Materials and Methods

### 2.1. Study Area and Period

**Figure 1:**
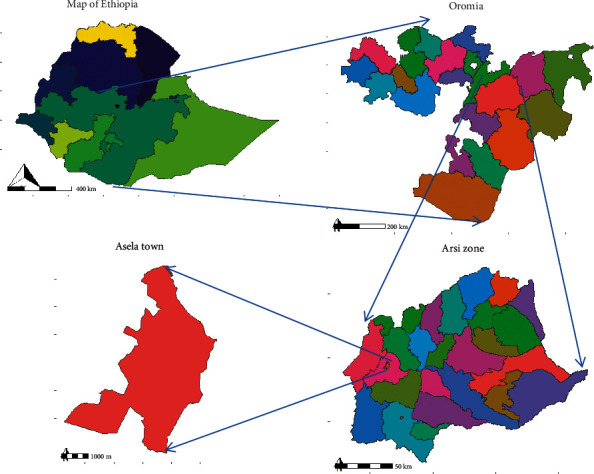
Location of the study site (https://www.mapsofindia.com/world-map/ethiopia/).

A prospective cross-sectional study was conducted at Asella town from March 1 to 30, 2022, Arsi Zone, Oromia Regional State. Asella is located 175 km southeast of Addis Ababa. The latitude and longitude of the town are 7° 57′ N 39° 7′ E with an elevation and total area of 2,430 meters and 4,623 hectares, respectively. The topography of the town is characterized as rugged and inclined. Asella town is mainly categorized as having highland climate conditions. According to a 2022 estimation, the total population of Asella town was 139,537 [18]. Consuming cow milk and milk products is common in Asella town that can be produced in the town or collected from the farmers and sold in the vendor's house. The study area is indicated with an arrow (Figure 1).

### 2.2. Sample Size Determination and Sampling Technique

The source populations were all raw milk vendors. The study populations were randomly selected milk vendors in Asella town during the study period. The sample size was determined using a single population proportion formula by taking the overall prevalence (21.3%) of *Salmonella* from the Oromia region [19], 48.7% coliform from Sudan [20], and 17.5% *Shigella* from Ethiopia [21]. During sample size determination, the following assumption was used: a 5% margin of error with a 95% confidence level, a correction formula for a population less than 10,000, and 10% for a nonresponse rate. Accordingly, the total sample size calculated was 210.

A systematic random sampling technique was used to select study participants using a list of vendors available at Asella town as the sampling frame. A total of 380 eligible milk vendors available at Asella town were divided by sample size (*n* = 210) to obtain an interval (*k* value = 2). One vendor was selected randomly from the first two using the lottery method. Data collection started with randomly selected vendors, and then, every 2^nd^ milk vendor was included.

### 2.3. Eligibility Criteria

Milk vendors who sold raw milk in Asella town during the study period and unpasteurized milk were included in the study. Milk vendors who were unable to give a response and were not willing to participate were excluded from the study.

### 2.4. Data Collection

Data was collected using a structured interviewer-administered questionnaire. The questionnaire was developed after reviewing similar studies [22–24]. A pretested structured questionnaire was administered through face-to-face interviews after obtaining written informed consent from each study participant. The questionnaire was used to assess the hygienic status, handling, processing, storage, and cleanness of the storage areas including environmental monitoring and neatness of the water as well as the customer service delivery area.

### 2.5. Sample Collection Transport and Processing

#### 2.5.1. Sample Collection

A 50 ml raw milk sample was aseptically collected from bulk milk containers of vendors. After adequately mixing, it was added into a clean and sterile leak-proof container (Falcon tube). The Falcon tubes containing the milk sample were labeled and placed into an icebox and then transported to the Asella Referral and Teaching Hospital laboratory for microbial analysis. The samples of raw milk were preserved in an icebox at ≤4°C and transported for microbiological examination within 4 hours of collection. All sample containers were labeled/marked immediately before and after the sample had been taken. The sample collected was kept in the refrigerator (2-8°C) before forwarding unless otherwise indicated.

### 2.6. Isolation and Identification of Coliforms

The milk samples were examined for the presence of coliform, *Shigella*, and *Salmonella* following standard techniques and procedures [25]. The samples were serially diluted up to 1 : 10^−5^ by transferring 1 ml of the milk into 9 ml of 0.1% peptone water for initial dilution. 1 ml of the diluted sample was transferred into 9 ml of peptone water, and the duplicate sample (1 ml) was poured using 15-20 ml violet red bile agar solution (VRBA) for serial dilution. After the medium was completely solidified, the surface of the medium was covered with a layer of the diluted sample and then incubated at 37°C for 24 hours. Colonies with characteristics of red-purple, 0.5 mm or greater in diameter, surrounded by a reddish halo were identified for further identification of coliforms [26]. The red-purple colonies were suspended in nutrient agar for further identification of each coliform based on their biochemical reaction. Triple sugar iron (TSI) agar, urea, citrate, mannitol fermentation, lysine iron agar, and sulfur indole motility (SIM) testing were used to identify the bacteria (Table 1).

### 2.7. Isolation of *Salmonella*

Buffered peptone water was used as a pre-enrichment medium, selenite broth as a selective enrichment medium, and XLD agar (xylose lysine deoxycholate agar) as a selective differential medium [27]. All suspected *Salmonella* colonies (having a slightly transparent zone of reddish color and a black center) were picked from the nutrient agar and inoculated into the biochemical test tubes for identification [23].

### 2.8. Isolation of Shigella

Specimens were plated directly on primary media: on Selenite F Broth (Mast Diagnostics, UK). For those negative samples on primary sold media, subculturing from enrichment broth to primary media was performed to improve the recovery of the isolates. All of the inoculated media were incubated at 37°C for 18-24 hours. The colorless SS agar and red on XLD agar colonies without black in the center were suspected of as *Shigella*. Kligler iron agar (KIA) was used for the differentiation of *Shigella* from other coliform bacteria. Suspected colonies were inoculated on a *Salmonella*-*Shigella* agar plate (Merck) and deoxycholate citrate agar (DCA) (Oxoid Ltd., UK) and incubated at 37°C for 24 hours. The change of a red butt to yellow and remaining the slope as red on KIA is confirmation of *Shigella* species [21].

### 2.9. Data Analysis

Data were checked for completeness and entered into Epi Info version 7.1 and exported to SPSS version 25 (IBM, New York, USA) for analyses. Descriptive statistics such as percentages and frequency distribution were used to describe/present bacterial isolates and related data. Binary logistic regression was used to assess the association between independent variables including sociodemographic and hygienic practice of the vendors and the outcome variable. Binary logistic regression was done, and variables with a *p* value < 0.25 were selected for multivariable analysis. Possibility of multicollinearity was checked before running multivariable logistic regression. Considering all indicators used to diagnose multicollinearity together, variables which had variance inflation factor (VIF) greater than 10 (1/(1–*R*^2^)), tolerance less than 0.1 (1–*R*^2^), condition index greater than 50 (or 30), eigenvalue less than 0.01, and proportion of variation greater than 0.8 (or 0.7) were excluded from multivariate analysis. The level of statistical significance was stated at *p* < 0.05.

### 2.10. Quality Control

Data quality was ensured using standardized data collection materials, pretesting of the questionnaires, proper orientation of data collectors before the start of data collection, and intensive supervision during data collection by the investigators. For laboratory analysis, preanalytical, analytical, and postanalytical stages of quality assurance incorporated in the standard operating procedures (SOPs) of the microbiology laboratory were strictly followed. Besides, a well-trained and experienced microbiologist was participating in the laboratory analysis procedure. Medium sterility was checked after preparation and incubating for 24 hours. Quality control strains such as *E. coli* (ATCC-25922), *Shigella flexneri* ATCC 12021, and *Salmonella Typhimurium* ATCC 14028 were obtained from the Ethiopian Public Health Institute (EPHI) to check the characteristics of the colony while growing respective media and biochemical tests.

### 2.11. Ethical Consideration

Ethical approval was obtained from the institutional review board (IRB) of Hawassa University College of Medicine and Health Sciences. Permission was obtained from study sites. Study participants were recruited after written informed consent was obtained.

## 3. Results

### 3.1. Sociodemographic Characteristics of the Study Participants

A total of 210 milk vendors were included in the study making a response rate of 100%. The median age of study participants was 32 years (IQR = 24-41 years). Two-thirds 58.6% of milk vendors were aged ≤35 years. Female participants accounted for 108 (55.6%) of the study participants. One hundred and thirty-seven (65.2%) of participants were at an elementary and secondary level of education. More than half (53.3%) of the participants were merchants (Table 2).

### 3.2. Hygienic Practice

More than three-fourths or 76.2% of vendors had a habit of washing teats whereas 120 (57.1%) did not wash teats with detergent. About 136 (64.8%) of vendors reported that there was a separate place for milking cows. Among the interviewed vendors, 133 (63.3%) of them practiced hand washing before milking a cow. Before selling the milk, 122 (58.2%) participants responded that they store milk at room temperature. About 166 (79.0%) of them stored milk in a plastic container. More than three-fourths (76.7%) of vendors did not test the milk for bacterial contamination and did not have a separate vending environment (Table 3).

### 3.3. Overall Percentage of Contamination of Milk

**Figure 2:**
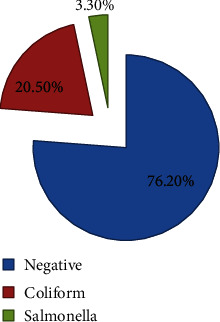
Bacterial isolates from raw milk among milk vendors at Asella town, Oromia, Ethiopia, 2022 (*N* = 210). Key: negative: no growth.

The overall percentage of bacterial contamination of milk was 23.8% (50/210) (95% CI: 18.1-29.5%). The predominant bacteria were coliform (43/210) 20.5% (95% CI: 15.2-26.2%), followed by *Salmonella* (7/210) 3.3% (95% CI: 1-5.7%) (Figure 2).

### 3.4. Percentage of Coliform

**Figure 3:**
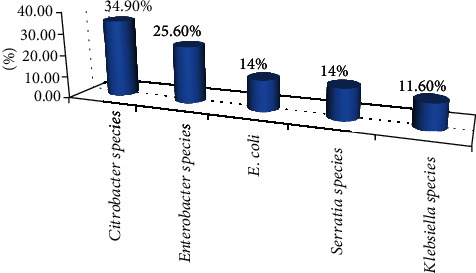
Coliform isolates from the raw milk at Asella town, Oromia, Ethiopia, 2022.

The majority of the coliform isolates from the raw milk were *Citrobacter* 15, 34.9%; *Enterobacter* 11, 25.6%; *E. coli* 6, 14%; *Serratia* 6, 14%; and *Klebsiella* 5, 11.6% (Figure 3).

### 3.5. Factors Associated with Bacterial Contamination of Raw Milk

In binary logistic regression, not having the habit of washing teats, not having the habit of washing teats with detergent, washing teats with only tap water, not having a separate place for milking, not washing hands before milking, not wearing separate cloth during milking, not having a practice of testing milk for bacterial contamination, not having separate vending environment, not sweeping vending environment, and not having separate waste disposal in the vending environment were candidate variables for multivariate analysis (*p* < 0.25). However, after fitting those variables into a multivariate logistic regression model, not having the habit of washing teats ((AOR = 77.3, 95% CI, 11.5-516.9), *p* < 0.0001), the habit of washing teats with only tap water ((AOR = 26.1, 95% CI, 5.5, 123.5), *p* < 0.0001), did not have separate cloth to be used during milking ((AOR = 39.98, 95% CI, 8.2, 196.07), *p* < 0.0001), not having a practice of testing milk for bacterial contamination ((AOR = 0.191, 95% CI, 0.04, 0.83), *p* = 0.027), and not having separate vending environment ((AOR = 8.4, 95% CI, 1.11, 63.4), *p* = 0.039) were significantly associated with bacterial contamination of raw milk (Table 4).

## 4. Discussion

Coliform, *Shigella*, and *Salmonella* are not only regarded as an indicator of fecal contamination but are more likely an indicator of poor hygiene and sanitary practices during milking and further handling.

In this study, 43 (20.5%) milk samples were contaminated with coliform (95% CI: 15.2-26.2). This finding is lower than studies from India (50%) [27], Iran (79%) [10], and the Khartoum State of Sudan (48.7%) [20]. The probable justification for the lower percentage of coliform contamination in the current study might be due to the nature of the study participants included. In the current study, only milk vendors were included unlike studies conducted in India, Iran, and Sudan where milk vendors, milk from shops, milk from dairy producers, and cafeterias were included. In addition, the decreased percentage may be attributed to the procedures being focused on traditional culture approaches instead of molecular approaches with high sensitivity.

The finding of the present study is higher than the study from the urban area of Algeria (12%) [25]. The possible justification for the higher percentage in this study could be a larger sample size unlike that of a study from the urban area of Algeria where only 20 raw milk samples were analyzed and poor hygienic handling practices and initial contamination from the unhygienic environment of vendors.

The present study showed a percentage of 3.3% of *Salmonella* from raw milk. This finding is lower as compared to studies from Bangladesh (45%) [6], Nigeria (46.7%) [28], Tanzania (37.33%) [29], Kenya (7.3%) [30], Egypt (15%) [31], Dire Dawa (18.75%) [32], and Addis Ababa (10.7%) [33]. However, the finding of the present study was higher than studies from Sebeta town of Ethiopia (0.7%) [9] and Addis Ababa (0.0%) [7]. Likewise, this study finding is consistent with studies from Rwanda (3.3%) [2]; Jigjiga, Somali (3.3%) [21]; and Holeta, Ethiopia (5.6%) [23]. The existence of such discrepancy is mainly due to variations in the study period, differences in laboratory method employed for isolation of *Salmonella*, and the difference in geographical location as well as hygiene practices of vendors and vending environment including environmental and seasonal factors. Besides this, it also indicates that the quality indicator of milk product is different from place to place.

In this study, no *Shigella* was detected in all samples examined. Unlike this study, *Shigella* was reported from raw milk in Jigjiga in Somalia at 17.5% [21] and in Kenya at 59% [30]. The existence of such difference might be attributed to milk vendors in Asella town having a better understanding of hygiene practices on personnel, maintaining the proper cold chain, and better hygiene practices in the milk vending environment, and laboratory methods employed for identification of *Shigella* may also bring these differences.

The finding of the present study revealed that the odds of bacterial isolation from raw milk increased by 77-fold among milk vendors that did not have a habit of washing cow teats as compared to those that did wash (*p* < 0.0001). This finding is consistent with studies conducted in eastern Tigray, Bahir Dar, and Addis Ababa, Ethiopia [17, 22, 23]. The possible justification for this association could be calf suckling facilitates the contamination of the milk from contaminated calves while milking. In addition, most milk vendors believe that the teats are washed or cleaned by the saliva of the calf, so it is not essential to clean the teats before milking. These significantly facilitated microbial contamination of milk because of cow's dung and infestation of the udder by flies.

This study also showed that milk vendors who had a habit of washing cow teats with tap water only had 26 times more likely higher odds of bacterial contamination of raw milk as compared to those who washed using detergents (*p* < 0.0001). The finding of this study was also supported by a study from Adigrat town that showed untreated groundwater used to wash the cow teat may have contributed to the high level of enteric bacteria contamination of milk [21, 22]. The possible justification for this association might be tap water is naturally existing water, and it contains plenty of microorganisms including pathogenic microbes that may contaminate the milk utensil; hence, the use of safe or boiled and portable clean water with detergent in washing milking equipment, hands, and udder is a good way to remove milk leftovers including pathogens which affect the microbiological safety of raw milk [23].

In this study, the odds of bacterial identification from raw milk increased by 39-fold among milk vendors who did not have separate cloth worn during milking as compared to those who did (*p* < 0.0001). This finding is concordant with a study from Adigrat town that they reported wearing separate clothes during milking is not practiced by milk collection centers and milk vendors, so poor hygienic condition of clothing contaminates milk while milking and selling [22].

In the present study, the odds of bacterial identification from raw milk were 81% less likely among milk vendors that did not undergo testing milk for bacterial contamination as compared to those that did (*p* = 0.027). This finding is parallel with a study from Kenya that quality control systems aimed at the prevention of defects, rather than their detection better in the prevention of milk contamination by milk-borne pathogens [34]. Moreover, this study also showed that the odds of bacterial identification from raw milk increased by 8-fold among vendors that did not have a separate vending environment as compared to those that did (*p* = 0.039). This finding was in agreement with a study from Adigrat town that showed no separate vending environment is suitable for microbial contamination of milk as a rise from the dung of cows reproduces and infects the milk as far as the barn is not apart from the vending environment [22].

## 5. Limitation of Study

The data were collected using an interviewer-administered method, so the responses were prone to social desirability biases. Due to resource constraints, microbiological analyses of all milk pathogens and antimicrobial resistance patterns were not considered in this study.

## 6. Conclusions

The result of this study identified that the overall percentage of bacterial contamination of milk was found to be high. A predominant type of bacteria was coliform followed by *Salmonella*. Not having the habit of washing teats, a habit of washing teat/milk utensils with only tap water, not having a separate cloth to be worn during milking, not having a practice of testing milk for bacterial contamination, and not having a separate vending environment were independent determinants of bacterial contamination of raw milk. Training programs on best practices for milk handling must be provided for milk vendors including increasing public awareness about the safety of raw milk. All milk vendors need to develop the habit of washing teats before milking, washing teat/milk utensils with detergent and tap water, wearing separate clothes while milking cows, and having separate vending environments. There should be adequate inspection of milk production facilities with microbiological controls of milk.

## Figures and Tables

**Table 1:** Biochemical used to identify the bacteria.

Biochemical tests	*Isolates*						
	*Klebsiella* spp.	*E. coli*	*Enterobacter* spp.	*Citrobacter* spp.	*Serratia* spp.	*Shigella* spp.	*Salmonella* spp.
Lactose	LF	LF	LF	LF	LF	NLF	NLF
Indole	Pos/Neg	Pos	Pos/Neg	Neg	Neg	Neg	Pos/Neg
Mannitol	MF	MF	MF	MF	MF	MF	MF
Urease	Pos	Neg	Neg	Pos/Neg	Pos	Neg	Pos/Neg
Citrate	Pos	Neg	Pos	Pos	Pos	Pos/Neg	Pos
H2S	Neg	Neg	Neg	Pos	Neg	Neg	Pos
Motility	Neg	Pos	Pos	Pos	Pos	Neg	Pos
Gas	Pos	Pos	Pos	Pos	Neg	Pos	Neg
LDC	Pos	Pos	Neg	Neg	Pos	Pos/Neg	Pos/Neg

Key: Pos: positive; Neg: negative; LDC: lysine decarboxylase; LF: lactose fermenter; NLF: nonlactose fermenter; MF: mannitol ferment; H2S: hydrogen sulfide.

**Table 2:** Sociodemographic characteristics of raw milk vendors at Asella town, Oromia, Ethiopia, 2022 (*N* = 210).

Variables	Categories	Frequency (%)
Age (in years)	≤35	123 (58.6)
	≥36	87 (41.4)

Gender	Male	102 (48.6)
	Female	108 (51.4)

Educational status	No formal education	32 (15.2)
	Elementary and secondary	137 (65.2)
	Degree and above	41 (19.5)

Occupation	Merchant	112 (53.3)
	Farmer	18 (8.6)
	Private worker	38 (18.1)
	Governmental	24 (11.4)
	Other	18 (8.6)

Others refer to housewives, daily labourers, and jobless.

**Table 3:** The hygienic practice of milk vendors at Asella town, Oromia, Ethiopia, 2022 (*N* = 210).

Variables	Categories	Frequency (%)
Technique of milking	Washing teat	166 (79.0)
	Calf sucking	44 (21.0)

The habit of washing teat	No	50 (23.8)
	Yes	160 (76.2)

The habit of washing teats with detergent	No	120 (57.1)
	Yes	90 (42.9)

The practice of washing teats with tap water only	No	140 (66.7)
	Yes	70 (33.3)

Use of towel for drying udder	Common towel	123 (58.6)
	Using pure hand	63 (30.0)
	Do not wash udder	24 (11.4)

The presence of a separate place for milking cow	No	74 (35.2)
	Yes	136 (64.8)

Storage method before selling milk	At room temperature	122 (58.1)
	Use of refrigerator	88 (41.9)

The practice of washing hands before milking	No	77 (36.7)
	Yes	133 (63.3)

The presence of separate cloth to be used during milking	No	87 (41.4)
	Yes	123 (58.6)

Type of utensils	Aluminum and stainless steel	31 (14.8)
	Plastic	166 (79.0)
	Clay spot	6 (2.9)
	Traditional equipment	7 (3.3)

Source of water for cleaning utensils	Tap water	41 (19.5)
	River	11 (5.2)
	Spring water	6 (2.9)
	Pipe water	152 (72.4)

The practice of testing milk for bacterial contamination	No	161 (76.7)
	Yes	49 (23.3)

The presence of a separate vending environment	No	49 (23.3)
	Yes	161 (76.7)

The practice of sweeping the vending environment	No	65 (31.0)
	Yes	145 (69.0)

The practice of using detergents while sweeping the vending environment	No	138 (65.7)
	Yes	72 (34.3)

The presence of separate waste disposal	No	90 (42.9)
	Yes	120 (57.1)

**Table 4:** Factors associated with bacterial contamination of raw milk among milk vendors at Asella town, Oromia, Ethiopia, 2022 (*N* = 210).

Variables	Category	Percentage (no. of Pos/total)	COR (95% CI)	*p* value	AOR (95% CI)	*p* value
Sex	Male	25.5 (26/102)	1.2 (0.6, 2.26)	0.578		
	Female	22.2 (24/108)	1	1		

Level of education	No formal education	21.9 (7/32)	1.36 (0.4, 4.3)	0.460		
	Elementary and secondary	26.3 (36/137)	1.7 (0.7, 4.2)	0.444		
	Degree and above	17.1 (7/41)	1	1		

Age (in years)	≤35	24.4 (30/123)	1.08 (0.56, 2.06)	0.814		
	>35	23.0 (20/87)	1	1		

The habit of washing teat	No	72.0 (36/50)	26.8 (11.7, 61)	<0.0001	77.3 (11.5,516)	<0.0001
	Yes	8.8 (14/160)	1	1	1	

Washing teat with detergent	No	37.5 (42/112)	6.75 (2.98, 15)	<0.0001	0.9 (0.17, 4.64)	0.895
	Yes	8.2 (8/98)	1	1	1	

Washing teat with only tap water	Yes	45.1 (32/71)	5.5 (2.79, 10.9)	<0.0001	26.1 (5.5, 123.5)	<0.0001
	No	12.9 (18/139)	1	1	1	

Separate place for milking cow	No	48.6 (36/74)	8.25 (4.0, 16.9)	<0.0001	1.1 (0.21, 5.37)	0.949
	Yes	10.3 (14/136)	1	1	1	

Storage method before selling milk	Room temperature	27.9 (34/122)	1.7 (0.889, 3.4)	0.104		
	Use of refrigerator	18.2 (16/88)	1	1		

Washing hands before milking	No	48.6 (36/77)	7.5 (3.7, 15.2)	0.002	3.5 (0.86, 14.02)	0.080
	Yes	10.3 (14/133)	1	1	1	

Separate cloth is worn during milking	No	46.0 (40/87)	9.6 (4.4, 20.8)	0.001	39.98 (8.2, 196.0)	<0.0001
	Yes	8.1 (10/123)	1	1	1	

The practice of testing milk for bacterial contamination	No	19.3 (31/161)	0.37 (0.19, 0.8)	0.005	0.19 (0.04, 0.83)	0.027
	Yes	138.8 (19/49)	1	1	1	

Separate vending environment	No	53.1 (26/49)	6.45 (3.2, 13.1)	<0.0001	8.4 (1.11, 63.4)	0.039
	Yes	14.9 (24/161)	1	1	1	

Sweeping vending environment	No	50.8 (33/65)	7.76 (3.8, 15.6)	<0.0001	0.3 (0.04, 2.08)	0.223
	Yes	11.7 (17/145)	1	1	1	

Separate waste disposal	No	34.4 (31/90)	2.79 (1.45, 5.4)	0.002	1.69 (0.337, 8.5)	0.523
	Yes	15.8 (19/120)	1	1	1	

1: reference category; COR: crude odd ratio; AOR: adjusted odd ratio.

## Data Availability

The datasets used and/or analyzed during the current study are available from the corresponding and main author on reasonable request.
